# SARA^speech^—Feasibility of automated assessment of ataxic speech disturbance

**DOI:** 10.1038/s41746-023-00787-x

**Published:** 2023-03-16

**Authors:** M. Grobe-Einsler, J. Faber, A. Taheri, J. Kybelka, J. Raue, J. Volkening, F. Helmhold, M. Synofzik, T. Klockgether

**Affiliations:** 1 German Center for Neurodegenerative Diseases (DZNE), Bonn, Germany; 2 Department of Neurology, University Hospital Bonn, Bonn, Germany; 3 PeakProfiling GmbH, Berlin, Germany; 4 Division of Translational Genomics for Neurodegenerative Diseases, Hertie-Institute for Clinical Brain Research and Center of Neurology, University Tübingen, Tübingen, Germany; 5 German Center for Neurodegenerative Diseases (DZNE), Tübingen, Germany

**Keywords:** Movement disorders, Spinocerebellar ataxia

## Abstract

Ataxias are a group of movement disorders that are characterized by progressive loss of balance, impaired coordination and speech disturbance, which together lead to markedly reduced quality of life. Speech disturbance is clinically diagnosed, but methods for objective assessment of severity are lacking. Using 71 sets of speech recordings from ataxia patients, we developed an automated classification system. With a tolerance of ±1 point, this classification system correctly predicted experts’ ratings of speech disturbance according to item 4 of the Scale for Assessment and rating of ataxia (SARA) in 80% of cases. We thereby demonstrate feasibility of computer-assisted voice analysis for automated assessment of severity of speech disturbance.

Ataxias are a heterogeneous group of movement disorders due to damage of the cerebellum and related brain structures. Clinical features include progressive loss of balance, impaired coordination and characteristic speech disturbance. The search for symptomatic and disease-modifying therapies for ataxias has not yielded medications with clear benefits, but recent advances in understanding of the disease mechanisms suggest promising avenues for novel therapy approaches which are currently tested in clinical trials. Speech disturbance is an important target symptom of new treatments. Clinically, it is characterized by imprecise articulation, irregular rhythm, and monotone speech pattern. Speech tempo is usually reduced but can abruptly accelerate with sudden increase in volume (explosive voice)^1^.

The Scale for the Assessment and Rating of Ataxia (SARA) is a widely used instrument to rate severity of ataxia^2^. Among the eight SARA items, item 4 is dedicated to speech. In this item, severity of ataxic speech disturbance is scored according to the degree of intelligibility (0: normal, (1) suggestion of speech disturbance, (2) Impaired speech but easy to understand, (3) Occasional words difficult to understand, (4) Many words difficult to understand, (5) Only single words understandable, (6) Speech unintelligible/anarthria). One of the three functional measures of the Spinocerebellar Ataxia Functional Index (SCAFI) is the PATA rate, which provides additional information on the speed of syllable repetition^3^.

Although impairment of speech strongly contributes to reduced quality of life of ataxia patients^4^, development of objective assessment methods employing computer-assisted voice analysis has only begun. Previous work characterized ataxic speech in distinctive ataxias, differentiated between healthy controls, pre-ataxic and ataxic patients and attempts were made to automate standardized speech tasks^5–7^. Given the prominence of speech disturbance in ataxia, computer-assisted analysis of speech allows an objective assessment, including features that are not accessible to the hearing of the human examiner. In this feasibility study, we aimed to develop an automated assessment of ataxic speech, SARA^speech^, and demonstrate its capacity to assess the severity of ataxic speech.

We analyzed 71 sets of speech recordings from 67 patients (42 male, 25 female) with mixed types of progressive degenerative ataxias. Mean age was 52 years (SD 14) and mean SARA score was 15.5 (SD 6). Median rating of SARA item 4 was 2 points (IQR: 1–3). For detailed description of the cohort, see Table 1. To investigate the potential of computer-assisted voice assessment to predict the magnitude of SARA item 4 score, machine learning methods were applied. In this approach, 117 prosodic and 24 lexical features extracted from the recordings were used. Prosodic features represent characteristics of the sound of the voice, such as the modulation of loudness, timbre, or fundamental frequency. Lexical features are, in general, based on textual information and focus on the intelligibility of single words in this study. Based on these features, 85% in the cross validated set (*z* = 5.78, *p* < 0.001, Fig. 1) and 80% 95% CI [0.5;1.0] in the hold-out set (Supplementary Fig. 1) of patients’ scores were predicted correctly with a ±1 tolerance in the k-fold cross-validation and test-set, respectively. The correlation (*Spearman*) of true and predicted labels was r = 0.42 (*p* < 0.001) and r = 0.57 95% CI [−0.19; 0.97].

**Table 1:** Patient characteristics.

	Age	Age of onset	Disease duration	SARA	Speech*
median	55	47	7	14.5	2
Q1, Q3	43, 60	36, 54	4, 13	12, 17	1, 3
mean	52	43	9	15.5	2
SD	14	16	7	6	1
95% CI	49, 56	39, 47	7, 11	14, 16.5	2, 2.5
Study population: *n* = 67 with 4 follow-ups (42 male, 25 female)					
Frequency of diagnoses: MSA-C (15), early onset ataxia (10), SCA3 (11), SCA6 (7), Friedreich’s ataxia (5), sporadic ataxia (5), SCA1 (4), SCA2 (3), hereditary spastic paraplegia with ataxia (3), SCA unknown (2), others** (6)					

*CANVAS* Cerebellar ataxia, neuropathy, bilateral vestibular areflexia syndrome, *CI* Confidence interval, *FXTAS* Fragile-X tremor ataxia syndrome, *MSA-C* Multi system atrophy (cerebellar type), *PSP-C* Progressive supra-nuclear palsy (cerebellar type), *SCA* Spinocerebellar ataxia, *SD* Standard deviation.

*Speech rating according to SARA Item 4.

**Alexander’s disease, CANVAS, FXTAS, PSP-C, SCA5, SCA8.

**Fig. 1:**
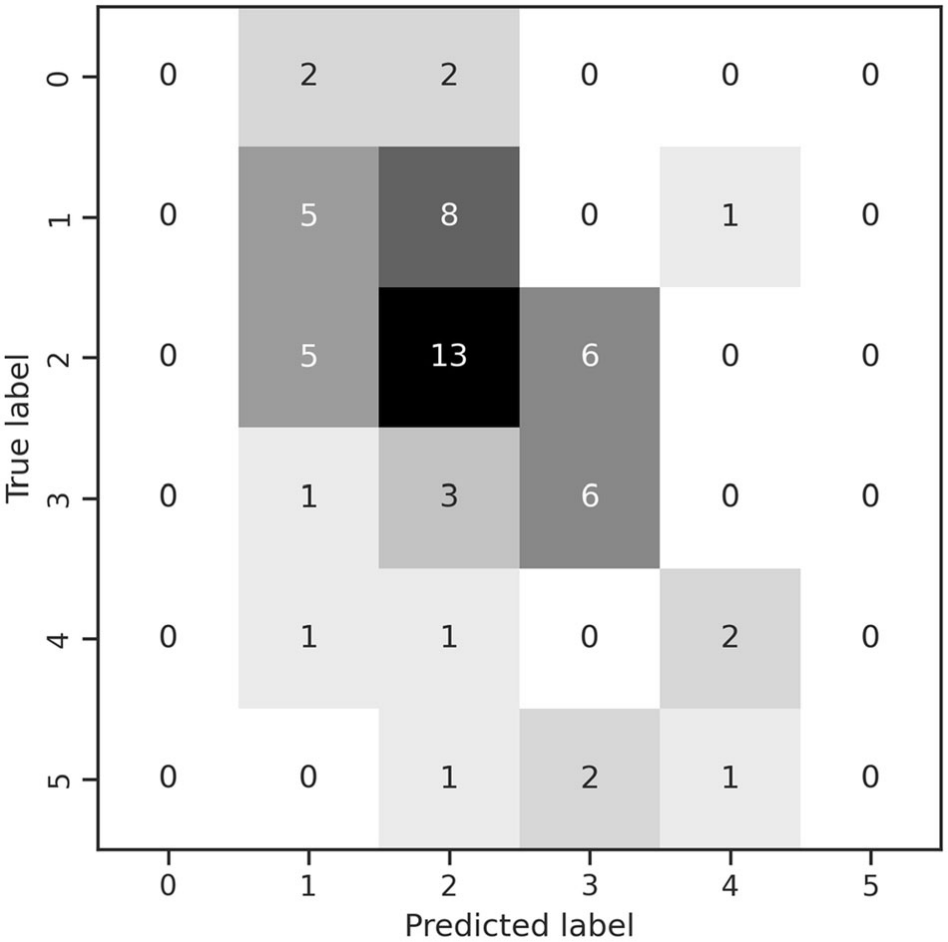
Results from SARA prediction. Confusion matrix of true and predicted SARA-scores based on SARA^speech^ machine learning approach using a broad set of voice features.

In the second step, feature importance of the most influencing prosodic and lexical characteristics was analyzed (Supplementary Fig. 2). Particularly, the type of the speech task as well as the different modules of voice features were considered. Five of the ten most influencing voice features were generated with the PATA task, three with the counting task, and two with free speech. Regarding the different modules of voice and speech features, Fourier tempogram was the most important feature. In addition, spectral features were found to be important, as seven out of ten of the most influencing features were of this type. In contrast, lexical features were solely important in free speech and represented less important feature importance for prediction analysis.

These results demonstrate feasibility of SARA^speech^ to predict the severity of speech disturbance according to SARA item 4. Previous studies analyzed batteries of voice features in recordings from patients with specific ataxias and healthy controls^6–8^. This led to improved characterization of speech impairment in these diseases and even enabled distinction between pre-ataxic mutation carriers, ataxic patients and healthy controls. These studies mainly focused on specific features, such as tempo and pitch variation. These can vary considerably even within a genetically homogeneous cohort^7^. In this study, we followed an alternative approach, i.e., to classify severity of speech disorder using established clinical scales, rather than characterizing it. Recordings are, therefore, not limited to patients from a homogeneous cohort. The used instrument was SARA item 4, which classifies the ataxic speech disorder without differentiating the various components, and assessment by experienced clinicians served as a blueprint for the development of the computer-based evaluation system, SARA^speech^. The use of established clinical scales promotes comparability to existing results from natural history studies. The used features are robust against interference and are therefore suitable for a wide range of applications, independent of the recording device.

Besides the use of a battery of open-source and proprietary voice features based on quantitative musicology, a novel procedure was applied to quantify the patient’s intelligibility. An automated speech-to-text system substituted the commonly used manual transcription and further provided an objective basis to evaluate the understandability of single words. Here, the intelligibility of each word was calculated and a general score for each participant was calculated. This score is a measure of how confident the model was in the transcription, i.e., how well the patient’s speech recording was understood. No target text is necessary for the calculations since the model specifies probabilities for letters and words based on German speech corpora at any time. The higher the probability, the more certain the model is, so the utterance was more understandable. The mean value of the probabilities of all words occurring in the transcript determines the confidence score. This intelligibility score represents the lexical features and provides a next step toward a fully automated, digital, and objective test instrument for measuring intelligibility in clinical practice.

PATA rate contributed more to the prediction than free speech and counting, suggesting that, in contrast to clinical rating, standardized speech tasks represent the strongest basis for computer-based analysis. In line with this, prosodic features were more important than lexical features.

The present results are proof-of-concept for the usefulness of computer-assisted voice analysis for speech assessment in ataxia and require an independent validation study to proof generalizability of the model. In particular, larger datasets including longitudinal recordings with more frequent representations of the scoring range are necessary to improve performance and bring the resultant algorithm to clinical applicability. Moreover, a higher number of patients will allow for a higher number of analyzed features and a hold-out set analysis with sufficient power. Additionally, possibly higher resolution of the graduation in between the point ranges of SARA item 4 scale could arise. Consequently, feature reduction and subsequent validation of most important voice and speech features in an independent dataset could be applied to further facilitate robustness of the model. Future studies should also consider questionnaire-based assessment of quality of life to correlate with severity of speech disturbance.

When fully developed and clinically applicable, SARA^speech^ will have an enormous potential for use in clinical routine, observational studies and interventional trials. Ease of use and independence of dedicated hardware allow for application by patients at home using a smartphone app. SARA^speech^ will be particularly important for the recording of fluctuations of ataxia severity, which are experienced by many patients and limit validity of assessments performed during single hospital visits^9^. The use of the SARA item 4 scale represents a first step towards fully automated ratings of SARA or the self-applied version SARA^home^ in combination with motion analysis of the remaining items^9^.

## Methods

### Participants

Participants were recruited during ongoing observational studies within the German Center for Neurodegenerative Diseases (DZNE) in Bonn. Inclusion criteria were clinical diagnosis of progressive degenerative ataxia and ability to comply with the study protocol. The study was approved by the ethics committee of the university hospital Bonn, Germany. All patients gave written informed consent prior to participation.

### Recordings

Voice recordings were part of a complete recorded SARA assessment using a Canon VIXIA HF G21. First, free speech was assessed. Participants were asked to respond to the following question: “Please, tell me something about your hobbies”. Consecutive questions were asked if the patient talked for <30 s (with reference to their answer, or by adding the second question: “How does a normal day look like for you?”). Second, patients counted from one to ten and back at normal speed and repeated the syllables PATA for 10 s, as fast and clear as possible.

### Reference rating

Consensus ratings of free speech recordings were obtained by three clinicians (MGE, TK, MS, JF). Recordings were excluded from technical analysis if no consensus ratings could be obtained, the set of recording was incomplete, or the patient had anarthria (corresponding to 6 points in the rating of SARA item 4).

### Pre-processing

Pre-processing steps included elimination of identifying data, amplitude magnitude normalization and denoising procedure by splitting the audio into speech and voiceless segments and subtracting the mean energy of the silent parts from the whole recording to minimize the amount of noise in the recording.

### Feature extraction

From the audio recordings, prosodic and lexical features were extracted. The prosodic features can be further divided into spectral features capturing the timbre of the voice as well as speech tempo and rhythm-based features. The spectral-features are derivatives of a short-time Fourier transformation of the original audio-signal with further processing following a proprietary procedure. The tempo and rhythm features were based on the onset-envelope^10^, from which the tempogram and Fourier tempogram were computed along with other dedicated speech tempo features^11^. The Fourier tempogram features were aggregated following a similar procedure to the spectral features that measure both the average and variability of rhythmicity in speech. The dedicated speech tempo features include the average word, syllable and sub-syllable speech tempo, by comparing peaks in the averaged tempogram in the respective time domains. Proprietary features were provided by PeakProfiling (Berlin, Germany). For the computation of basic audio-representations (spectrogram, onset-envelope, tempogram and Fourier tempogram) the *Python* implementation of *librosa* was used^12^. The lexical features were extracted using *deep-speech*^13^, an open-source speech-to-text system for English and Mandarin, developed by *Mozilla*. The model was fine-tuned to the German language leveraging the German subset of the *CommonVoice* dataset^14,15^. From the pre-processed audio recordings, the averaged detected word count, real-word count and word duration were extracted for each of the recordings. The distinction between detected and real words was achieved using the German version of the *pyspellchecker* library^16^. Additionally, the confidence score of the fine-tuned model outputted while transcribing the recordings was exported as a feature capturing the overall intelligibility of the patient. An overview of open-source features that were further developed and used for analysis is attached (Supplementary Table 1).

### Machine learning

For each of the 71 sets of speech recordings a total of 141 features were extracted, decomposed into 117 prosodic and 24 lexical features. Combined with the SARA item 4 as target, the data basis for a supervised machine learning task is obtained. Due to the small sample size a 6-fold cross-validation scheme was applied for model selection and fine-tuning. To ensure generalization of the best model, a gradient-boosted tree ensemble as implemented by *xgboost*^17^ was trained on all folds of the cross-validation and tested on the independent hold-out set^18^ as shown in Supplementary Fig. 3. Metrics are reported separately for the pooled validation set (*n* = 60), containing the validation sets of the folds 1–6, as well as the hold-out set (*n* = 10). These included the *Spearman* rank correlation coefficient for the predicted with the actual SARA item 4 labels, as well as the accuracy and the probability of predicting the correct label with a ±1 tolerance. In order to analyze the importance of the features, fold by fold for each feature the average gain per tree in which this specific feature was used was calculated. Subsequently, the mean values were taken over all cross-validation folds. Statistical inference was performed using two-sided hypothesis tests on the cross validation set and by reporting the 95% confidence intervals of bootstrapped metrics in the test set.

### Reporting summary

Further information on research design is available in the Nature Research Reporting Summary linked to this article.

## Supplementary information

Supplementary Material

Reporting Summary

## Data Availability

The datasets generated during and/or analyzed during the current study are not publicly available due to identifying character of speech recordings. Other data is available from the corresponding author on reasonable request.

## Code availability

All open source libraries used in this study are publicly available. Further information is available in the Nature Research Reporting Summary to this article. Non-open source information is available from the corresponding author upon reasonable request.

## References

[1] Ackermann H, Ziegler W (1992). Die zerebelläre Dysarthrie-eine Literaturübersicht. Fortschr. Neurol. Psychiatr..

[2] Schmitz-Hübsch T (2006). Scale for the assessment and rating of ataxia: development of a new clinical scale. Neurology.

[3] Schmitz-Hübsch T (2008). SCA Functional Index: a useful compound performance measure for spinocerebellar ataxia. Neurology.

[4] Schmahmann JD, Pierce S, MacMore J, L’Italien GJ (2021). Development and validation of a patient-reported outcome measure of ataxia. Mov. Disord. Off. J. Mov. Disord. Soc..

[5] Mueller A (2021). Digital endpoints for self-administered home-based functional assessment in pediatric Friedreich’s ataxia. Ann. Clin. Transl. Neurol..

[6] Vogel AP (2020). Features of speech and swallowing dysfunction in pre-ataxic spinocerebellar ataxia type 2. Neurology.

[7] Vogel AP (2017). Voice in Friedreich ataxia. J. Voice. Off. J. Voice. Found..

[8] Vogel AP (2018). Coordination and timing deficits in speech and swallowing in autosomal recessive spastic ataxia of Charlevoix-Saguenay (ARSACS). J. Neurol..

[9] Grobe-Einsler M (2021). Development of SARAhome, a new video-based tool for the assessment of ataxia at home. Mov. Disord. Off. J. Mov. Disord. Soc..

[10] Böck, S. & Widmer, G. Maximum filter vibrato suppression for onset detection. In *Proceedings of the 16th International Conference in Digital Audio Effects (DAFx)*.

[11] Grosche, P., Müller, M., & Kurth, F. *Cyclic tempogram—a mid-level tempo representation for music signals* (2010).

[12] McFee, B. et al. librosa: Audio and Music Signal Analysis in Python. In *Proceedings of the 14th Python in Science Conference* (SciPy2015), pp. 18–24.

[13] Dario, A. et al. Deep speech 2: End-to-end speech recognition in english and mandarin. *International Conference on Machine Learning*, 173–182 (2016).

[14] Ardila, R. et al. Common Voice: A Massively-Multilingual Speech Corpus, 2019.

[15] Weiss K, Khoshgoftaar TM, Wang D (2016). A survey of transfer learning. J. Big Data.

[16] GitHub. GitHub - barrust/pyspellchecker: Pure Python Spell Checking http://pyspellchecker.readthedocs.io/en/latest. Available at https://github.com/barrust/pyspellchecker (2022).

[17] Chen, T. & Guestrin, C. XGBoost. In *Proceedings of the 22nd ACM SIGKDD International Conference on Knowledge Discovery and Data* Mining, edited by B. Krishnapuram (ACM, New York, NY, 2016), pp. 785–794.

[18] Rao, R. B., Fung, G. & Rosales, R. On the dangers of cross-validation. An Experimental Evaluation. In *Proceedings of the 2008 SIAM International Conference on Data Mining*, edited by C. Apte, H. Park, K. Wang & M. J. Zaki (Society for Industrial and Applied Mathematics, Philadelphia, PA, 04242008), pp. 588–596.

