# Supplementary material for: SARAspeech—Feasibility of automated assessment of ataxic speech disturbance
Source: NPJ Digit Med. 2023 Mar 16;6:43. doi: 10.1038/s41746-023-00787-x (PMC10020430; doi:10.1038/s41746-023-00787-x)
Supplement: Supplementary file 1 — Supplementary Material [file 41746_2023_787_MOESM1_ESM.pdf]

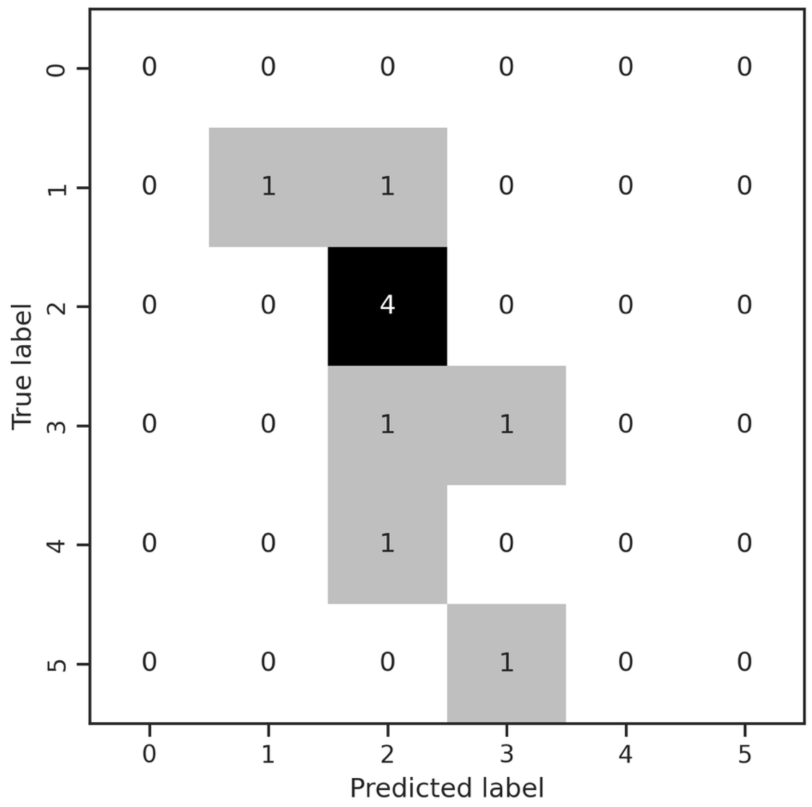

Supplementary Figure 1: Confusion matrix of true and predicted SARA-scores in an additional hold-our set based on SARA^{speech} machine learning approach using a broad set of voice features.

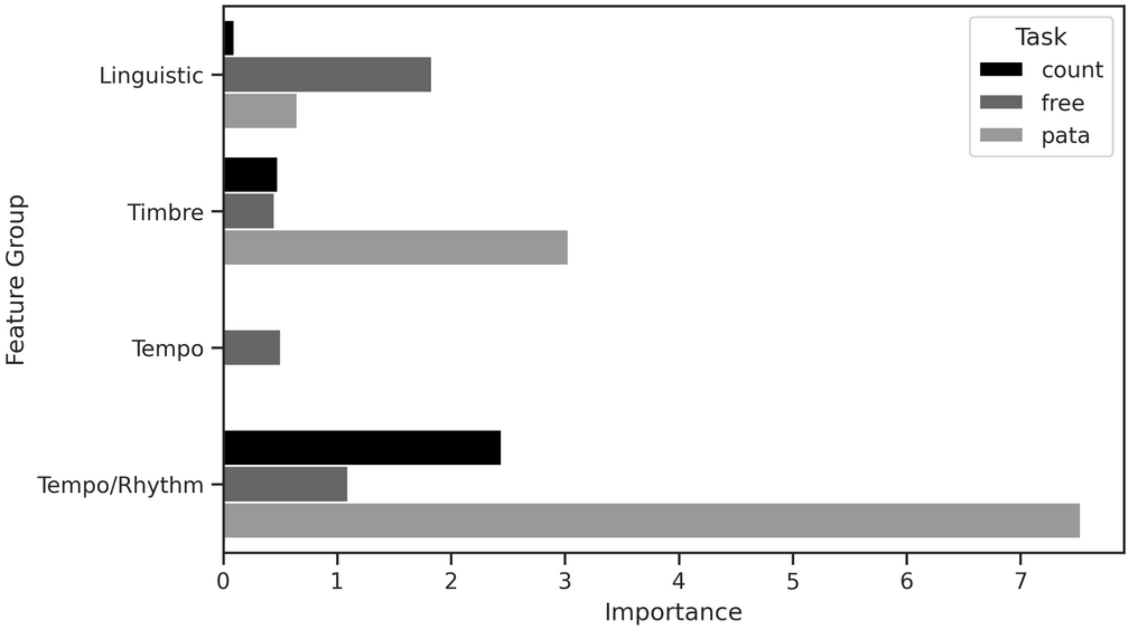

Supplementary Figure 2: Top ten voice and speech features contributing to prediction analysis results depending on specific speech task.

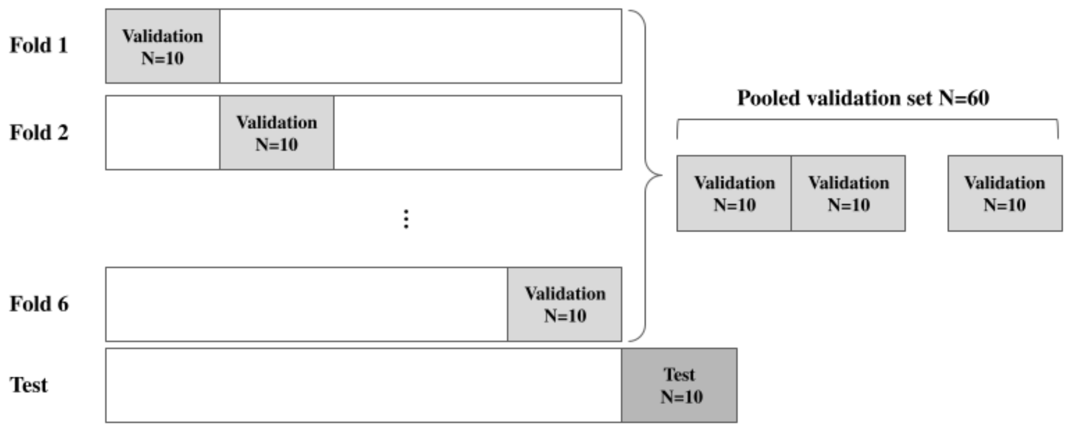

Supplementary Figure 3: Performance of the trained SARA^{speech} system was evaluated in a 6-fold cross validation scheme with an additional hold out set.

Domain	Name	Capturing	Basis	Implementation of Basis	Comment
**Paralinguistic**	Spectral	Timbre	STFT-Spectrogram	librosa.stft	Further developed
	Tempo	Speech Tempo	(Fourier) Tempogram	librosa.feature.tempogram librosa.feature.fourier_tempogram	Further developed
	Rhythm	Speech Rhythmicity, Variability	Onset-Envelope	librosa.onset.onset_strength	Further developed
**Linguistic**	Pronunciation	Intelligibility Speech Tempo	Speech-to-text	sttWithMetadata	- Count of words<br>- Count of real-words<br>- Confidence score (intelligibility)<br>- Duration of words

Supplementary Table 1: Open-source features used for further development and analysis.
