# Supplementary material for: SARAspeech—Feasibility of automated assessment of ataxic speech disturbance
Source: NPJ Digit Med. 2023 Mar 16;6:43. doi: 10.1038/s41746-023-00787-x (PMC10020430; doi:10.1038/s41746-023-00787-x)
Supplement: Supplementary file 2 — Reporting Summary [file 41746_2023_787_MOESM2_ESM.pdf]

nature portfolio

Corresponding author(s): Dr. Marcus Grobe-Einlser

Last updated by author(s): Jan 30, 2023

Reporting Summary

Nature Portfolio wishes to improve the reproducibility of the work that we publish. This form provides structure for consistency and transparency in reporting. For further information on Nature Portfolio policies, see our Editorial Policies and the Editorial Policy Checklist.

Statistics

For all statistical analyses, confirm that the following items are present in the figure legend, table legend, main text, or Methods section.

n/a	Confirmed	
☐	☒	The exact sample size (*n*) for each experimental group/condition, given as a discrete number and unit of measurement
☐	☒	A statement on whether measurements were taken from distinct samples or whether the same sample was measured repeatedly
☐	☒	The statistical test(s) used AND whether they are one- or two-sided<br>*Only common tests should be described solely by name; describe more complex techniques in the Methods section.*
☒	☐	A description of all covariates tested
☐	☒	A description of any assumptions or corrections, such as tests of normality and adjustment for multiple comparisons
☐	☒	A full description of the statistical parameters including central tendency (e.g. means) or other basic estimates (e.g. regression coefficient) AND variation (e.g. standard deviation) or associated estimates of uncertainty (e.g. confidence intervals)
☐	☒	For null hypothesis testing, the test statistic (e.g. *F*, *t*, *r*) with confidence intervals, effect sizes, degrees of freedom and *P* value noted<br>*Give P values as exact values whenever suitable.*
☒	☐	For Bayesian analysis, information on the choice of priors and Markov chain Monte Carlo settings
☒	☐	For hierarchical and complex designs, identification of the appropriate level for tests and full reporting of outcomes
☐	☒	Estimates of effect sizes (e.g. Cohen's *d*, Pearson's *r*), indicating how they were calculated

Our web collection on statistics for biologists contains articles on many of the points above.

Software and code

Policy information about availability of computer code

Data collection	Recordings were obtained during study visits with no software involved.
Data analysis	For our analyses we used Python and some open source libraries. More specifically, we used pandas v1.2.1 and numpy v1.20.0 for handling the data and statistical analyses. The spectral feature generation was mainly based on librosa v0.8.0 and the linguistic feature generation on deep-speech v0.9. Further processing steps followed using numpy and scipy v1.6. The final classifications were prepared with scikit-learn v0.24.1 and carried out with xgboost v1.6.1.<br>During cross-validation we tried xgboost with different parameter settings. We varied the number of parallel trees [1, 3, 5], the maximal depth of each tree [5, 10, 15] and the learning rate [0.1, 0.3, 0.5].

For manuscripts utilizing custom algorithms or software that are central to the research but not yet described in published literature, software must be made available to editors and reviewers. We strongly encourage code deposition in a community repository (e.g. GitHub). See the Nature Portfolio guidelines for submitting code & software for further information.

Data

Policy information about availability of data

All manuscripts must include a data availability statement. This statement should provide the following information, where applicable:
- Accession codes, unique identifiers, or web links for publicly available datasets
- A description of any restrictions on data availability
- For clinical datasets or third party data, please ensure that the statement adheres to our policy

The datasets generated during and/or analyzed during the current study are not publicly available due to identifying character of speech recordings. Other data is available from the corresponding author on reasonable request.

Human research participants

Policy information about studies involving human research participants and Sex and Gender in Research.

Reporting on sex and gender	n.a.
Population characteristics	patient characteristics are provided in the manuscript Table 1 in the manuscript.
Recruitment	patients were recruited during consecutive study visits within the German Center for Neurodegenerative Diseases in Bonn, Germany.
Ethics oversight	Ethics committee of the university hospital Bonn, Germany

Note that full information on the approval of the study protocol must also be provided in the manuscript.

Field-specific reporting

Please select the one below that is the best fit for your research. If you are not sure, read the appropriate sections before making your selection.

☒ Life sciences ☐ Behavioural & social sciences ☐ Ecological, evolutionary & environmental sciences

For a reference copy of the document with all sections, see nature.com/documents/nr-reporting-summary-flat.pdf

Life sciences study design

All studies must disclose on these points even when the disclosure is negative.

Sample size	Sample size was limited due to rarity of the disease. Maximum available number of recordings were included in the study.
Data exclusions	Consensus ratings of free speech recordings were obtained by three clinicians (MGE, TK, MS, JF). Recordings were excluded from technical analysis if no consensus ratings could be obtained, the set of recording was incomplete, or the patient had anarthria (corresponding to 6 points in the rating of SARA item 4).
Replication	All non-deterministic procedures of our classification pipieline have been seeded to ensure reproducability
Randomization	Assignment to train and test-sets were performed randomly
Blinding	not blinded

Reporting for specific materials, systems and methods

We require information from authors about some types of materials, experimental systems and methods used in many studies. Here, indicate whether each material, system or method listed is relevant to your study. If you are not sure if a list item applies to your research, read the appropriate section before selecting a response.

Materials & experimental systems

n/a	Involved in the study
☒	☐ Antibodies
☒	☐ Eukaryotic cell lines
☒	☐ Palaeontology and archaeology
☒	☐ Animals and other organisms
☒	☐ Clinical data
☒	☐ Dual use research of concern

Methods

n/a	Involved in the study
☒	☐ ChIP-seq
☒	☐ Flow cytometry
☒	☐ MRI-based neuroimaging
